# Supplementary material for: Coupling water fluxes with cell wall mechanics in a multicellular model of plant development
Source: PLoS Comput Biol. 2019 Jun 20;15(6):e1007121. doi: 10.1371/journal.pcbi.1007121 (PMC6605655; doi:10.1371/journal.pcbi.1007121)
Supplement: S1 Table — (PDF) [file pcbi.1007121.s001.pdf]

List of main notations

Here is the list of the main variables and parameters used in the article. Variables and parameters with a subscript (for instance P_i or ε_k^e) are relative to a given cell or edge.

Table S1: List of main notations: variables and parameters.

Name	Units	Description	First appearance
P	MPa	Hydrostatic pressure / Turgor	Eq. (1)
π	MPa	osmotic pressure	Eq. (2)
Ψ	MPa	Hydric potential	Eq. (2)
$\dot{\gamma}$	s^{-1}	Relative growth rate	Eq. (3)
$\dot{\varepsilon}$	s^{-1}	Strain rate	Eq. (1)
ε^e	Dimensionless	Elastic deformation	Eq. (7)
V	m^3	Cell volume	Eq. (2)
A	m^2	Cell area	Eq. (2)
l	m	Cell edge length	Eq. (1)
w	m	Thickness of the walls	Fig. 1
h	m	Height of the cells	Fig. 1
ϕ^w	$MPa^{-1} \cdot s^{-1}$	Effective wall extensibility (Lockhart/ortega model)	Eq. (1)
Φ^w	$MPa^{-1} \cdot s^{-1}$	Intrinsic wall extensibility	Eq. (7)
$\bar{E}$	MPa	Effective elastic modulus (Lockhart/ortega model)	Eq. (1)
E	MPa	Intrinsic elastic modulus	Eq. (7)
P^M	MPa	Power of the osmotic pump	Eq. (3)
P^Y	MPa	Threshold pressure	Eq. (1)
ε^Y	Dimensionless	Threshold elastic deformation	Eq. (7)
L^a	$m \cdot MPa^{-1} \cdot s^{-1}$	Water conductivity with external source	Eq. (2)
ϕ^a	$MPa^{-1} \cdot s^{-1}$	Quantifies the cells' ability to absorb water from the source	Eq. (3)
L^s	$m.MPa^{-1} \cdot s^{-1}$	Water conductivity between cells	Eq. (8)
ϕ^s	$MPa^{-1} \cdot s^{-1}$	Quantifies the cells' ability to exchange water with each other	Eq. (8)
$\dot{\gamma}^*$	s^{-1}	Relative growth rate, solution of the Lockhart model	Eq. (6)
P^*	MPa	Hydrostatic pressure / Turgor, solution of the Lockhart model	Eq. (4)
α^a	Dimensionless	Flux *vs* wall synthesis limitation to growth	Eq. (5)
α^s	Dimensionless	Flux with source *vs* fluxes between cells	Eq. (9)
