# Supplementary material for: Coupling water fluxes with cell wall mechanics in a multicellular model of plant development
Source: PLoS Comput Biol. 2019 Jun 20;15(6):e1007121. doi: 10.1371/journal.pcbi.1007121 (PMC6605655; doi:10.1371/journal.pcbi.1007121)
Supplement: S1 Text — The relation between turgor P and stress σ is derived from the mechanical equilibrium and the time-dependent solution is explicitely computed. (PDF) [file pcbi.1007121.s002.pdf]

Supplementary information for the article: Coupling water fluxes with cell wall mechanics in a multicellular model of plant development.

Calculations for Lockhart-Ortega models

Ibrahim Cheddadi, Michel Génard, Nadia Bertin, Christophe Godin

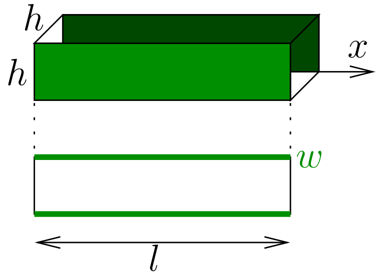

Figure A: Geometrical parameters of Lockhart-Ortega models: perspective view of the elongating cell and view from top. Geometrical parameters: height and width h, length l for the cell, thickness w for the walls. The two faces orthogonal to the x axis are refered to as base faces while the two faces in green are refered to as lateral faces.

The equations of cell wall elongation (Eq. (1) in main text) and of water uptake (Eq. 2 in main text) can be linked thanks to the geometry of the cell and the mechanical equilibrium. In this article we describe cells as 2D polygons that are given a 3D extension with a height h (see Fig. 1 in main text and Fig. A).

In the case of the Lockhart/Ortega model, the cells are elongating rectangles of length l in the elongating direction and width h. Therefore, the cell volume is $V = h^2 l$ and we find that the relative growth rate of the cell is equal to the strain rate of the walls:

$$\dot{\gamma} = \frac{1}{V}\frac{\mathrm{d}V}{\mathrm{d}t} = \frac{1}{l}\frac{\mathrm{d}l}{\mathrm{d}t} = \dot{\varepsilon}. \tag{S1}$$

Then, we consider the balance of forces on the base faces (see Fig. A for the nomenclature); their area is $h \times h$ and they are submitted to a total pressure force Ph^2 in the direction of the main axis of the cell, balanced by the tension from the lateral walls. Let σ be the common (scalar) stress in the walls; the wall thickness is w so their cross section is $h \times w$ and therefore they each exert a force σhw on the base faces. To be coherent with the bidimensional model we propose, we consider that the top and bottom lateral faces bear no stress and the balance of forces leads to

$$Ph^2 = 2\sigma hw$$

and therefore $P = 2\frac{w}{h}\sigma$. Finally, thanks to this equation and the identity (S1), the Lockhart-Ortega model (Eqs. (1), (3) in main text) is reduced to the following differential equation for P:

$$\frac{1}{\bar{\bar{E}}}\frac{\mathrm{d}P}{\mathrm{d}t} + \phi^w (P - P^Y)_+ = \phi^a \left(P^M - P\right), \tag{S2}$$

where $\phi^a = \frac{AL^a}{V}$ has been introduced in the main text; in order to keep the calculations as simple as possible, Lockhart made the assumption that the area of the base faces is negligeable compared to the area $A = 2hl$ of the lateral faces (see Fig. A). Note that the cell volume is $V = h^2 l$ and therefore the ratio $A/V = 2/h$ is constant.

Let's study the transient behaviour of equation (S2), from an initial condition $P(t = 0) = 0$:

- Elastic regime: first, P is below P^Y and the plastic rate is zero; (S2) becomes

$$\lambda^a \frac{\mathrm{d}P}{\mathrm{d}t} + P = P^M,$$

where $\lambda^a = \frac{1}{\phi^a \bar{E}}$ is a characteristic time. The solution is

$$P = P^M(1 - \exp(-t/\lambda^a)).$$

The relative growth rate is

$$\dot{\gamma} = \phi^a P^M \exp(-t/\lambda^a).$$

- Plastic regime: the plastic regime starts when $P = P^Y$, at $t^0 = \lambda^a \log\left(\frac{P^M}{P^M - P^Y}\right)$. The equation (S2) becomes:

$$\frac{1}{\bar{E}} \frac{\mathrm{d}P}{\mathrm{d}t} + (\phi^a + \phi^w)P = \phi^a P^M + \phi^w P^Y,$$

and equivalently

$$\lambda^{aw} \frac{\mathrm{d}P}{\mathrm{d}t} + P = \alpha^a P^M + (1 - \alpha^a)P^Y,$$

where $\lambda^{aw} = \frac{1}{(\phi^a + \phi^w)\bar{E}}$ is a characteristic time. The solution is

$$P = \alpha^a P^M + (1 - \alpha^a)P^Y - \alpha^a(P^M - P^Y)\exp((t^0 - t)/\lambda^{aw}), \tag{S3}$$

$$\dot{\gamma} = \frac{\phi^a \phi^w}{\phi^a + \phi^w}(P^M - P^Y) - \frac{(\phi^a)^2}{\phi^a + \phi^w}(P^M - P^Y)\exp((t^0 - t)/\lambda^{aw}). \tag{S4}$$

The stationnary solution is

$$P^* = \alpha^a P^M + (1 - \alpha^a)P^Y \tag{S5}$$

$$\dot{\gamma}^* = \frac{\phi^a \phi^w}{\phi^a + \phi^w}(P^M - P^Y). \tag{S6}$$
