# Supplementary material for: Coupling water fluxes with cell wall mechanics in a multicellular model of plant development
Source: PLoS Comput Biol. 2019 Jun 20;15(6):e1007121. doi: 10.1371/journal.pcbi.1007121 (PMC6605655; doi:10.1371/journal.pcbi.1007121)
Supplement: S2 Text — The relation between turgor P and stress σ is derived from the mechanical equilibrium as a function of cell size R and number of edges n. The time-dependent solution is numerically computed. (PDF) [file pcbi.1007121.s003.pdf]

Supplementary information for the article: Coupling water fluxes with cell wall mechanics in a multicellular model of plant development.

Calculations for a single polygonal cell

Ibrahim Cheddadi, Michel Génard, Nadia Bertin, Christophe Godin

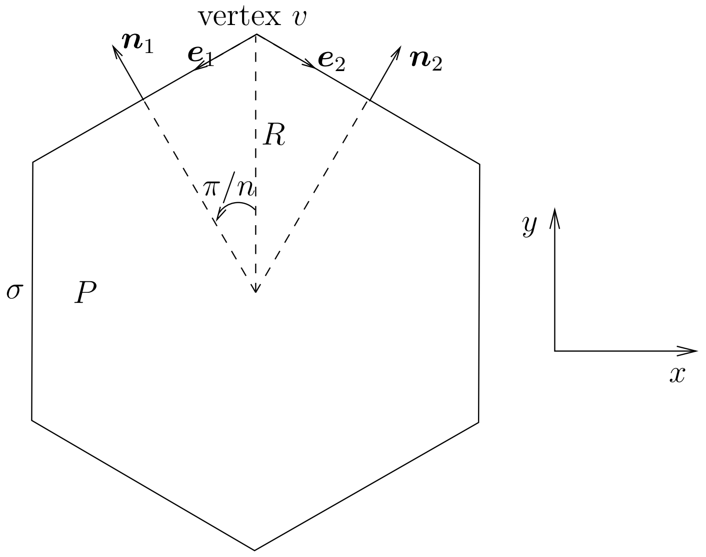

Figure A: Geometrical parameters for a polygonal cell (first extension of Lockhart model).

We consider a regular convex polygon of radius R with n edges that represents a cell.

Mechanical equilibrium. Let σ be the stress in the walls and P the pressure inside the cell; the outside pressure is set to zero. The length of the edges is $2R\sin(\pi/n)$, and the walls are given a height h and a thickness w; therefore the stresses are exerted on a surface hw; the contribution of pressure on vertex v is $\frac{1}{2}P2hR\sin(\pi/n)(\boldsymbol{n}_1+\boldsymbol{n}_2)$. Therefore, the balance of forces on vertex v writes:

$$\frac{1}{2}P2hR\sin(\pi/n)(\boldsymbol{n}_1+\boldsymbol{n}_2)+\sigma hw(\boldsymbol{e}_1+\boldsymbol{e}_2)=0.$$

The normal vectors are

$$\boldsymbol{n}_1=(-\sin(\pi/n),\cos(\pi/n)) \quad \text{and} \quad \boldsymbol{n}_2=(\sin(\pi/n),\cos(\pi/n)).$$

The tangent vectors are

$$\boldsymbol{e}_1=(-\cos(\pi/n),-\sin(\pi/n)) \quad \text{and} \quad \boldsymbol{e}_2=(\cos(\pi/n),-\sin(\pi/n)).$$

By symetry, the x component of the resulting force is zero; the projection of the balance of forces on y axis yields

$$2PhR\sin(\pi/n)\cos(\pi/n)-2\sigma hw\sin(\pi/n)=0,$$

and

$$P = \frac{w}{R\cos(\pi/n)}\sigma. \tag{S7}$$

When $n \to \infty$, $\cos(\pi/n) \to 1$ and we recover the Laplace law.

Flux equation. The surface of the polygon is

$$S_n = n \times 2R\sin(\pi/n)R\cos(\pi/n)/2 = R^2 n\sin(\pi/n)\cos(\pi/n).$$

The volume of the cell is $V = S_n h$, so the volume variation is

$$\frac{\mathrm{d}V}{\mathrm{d}t} = 2hR\frac{\mathrm{d}R}{\mathrm{d}t}n\sin(\pi/n)\cos(\pi/n).$$

The perimeter of the polygon is $n \times 2R\sin(\pi/n)$ so the lateral area of the cell is

$$A = 2nhR\sin(\pi/n).$$

Note that the ratio A/V is not constant:

$$\frac{A}{V} = \frac{2}{R\cos(\pi/n)}.$$

Finally, the flux equation writes

$$2hR\frac{\mathrm{d}R}{\mathrm{d}t}n\sin(\pi/n)\cos(\pi/n) = n2hR\sin(\pi/n)L(P^M - P),$$

which yields

$$\frac{\mathrm{d}R}{\mathrm{d}t} = \frac{L}{\cos(\pi/n)}(P^M - P) \tag{S8}$$

Wall rheology. Let ε^e be the elastic deformation of the walls; it is related to the stress by the constitutive equation $\sigma = E\varepsilon^e$ where E is the elastic modulus. The length of the edges is $l = 2R\sin(\pi/n)$ and therefore the strain rate of the edges is $\frac{1}{l}\frac{\mathrm{d}l}{\mathrm{d}t} = \frac{1}{R}\frac{\mathrm{d}R}{\mathrm{d}t}$. The rheological behaviour of the walls is given by

$$\frac{1}{R}\frac{\mathrm{d}R}{\mathrm{d}t} = \frac{\mathrm{d}\varepsilon^e}{\mathrm{d}t} + \Phi^w E\max(0, \varepsilon^e - \varepsilon^Y), \tag{S9}$$

or equivalently

$$\frac{1}{R}\frac{\mathrm{d}R}{\mathrm{d}t} = \frac{1}{E}\frac{\mathrm{d}\sigma}{\mathrm{d}t} + \Phi^w\max(0, \sigma - \sigma^Y), \tag{S10}$$

where ε^Y (resp. σ^Y) is a yield elastic deformation (resp. stress).

Numerical results. The problem to solve is reduced to a set of two differential equations. It is numerically solved with the `odeint` routine from the `python` library `scipy`.

We study the growth of a hexagonal cell ($n = 6$) growing from an initial state where the elastic deformation of the walls is set to the threshold value, in order to bypass the pure elastic regime; computations are run over a long time scale. We want to study how this models compares to Lockhart-Ortega when the relative importance of fluxes and wall synthesis varies; to this end, we run three simulations with $\alpha^a = 0.1, 0.5, 0.9$. Let $R_0 = 10\mu$m be the initial radius of the cell, then $P^Y = \frac{w}{R_0\cos(\pi/6)}E\varepsilon^Y$ is a representative value for the yield turgor of a hexagonal cell. The value $\varepsilon^Y = 0.1$ is chosen accordingly to experimental observations where wall deformations can be of the order of 10%; then we choose E such that $P^Y = 0.5$ MPa, which sets an order of magnitude for the initial turgor of the cell, close to observed experimental data. We choose $P^M = 0.7$ MPa so that it is above P^Y. Finally, we can use the Lockhart's prediction (Appendix 1, eq. S6) as an order of magnitude of the relative growth rate; we choose $\dot{\gamma}^* = 2\% \cdot \mathrm{h}^{-1}$. Then, a given value of α^a (evaluated with the initial area of the cell) sets a unique value of L^a and ϕ^w.

At the onset of the simulation, walls start to extend irreversibly and plastic growth occurs. Figs. Ba,c show that the volume increases faster for large values of α^a, although we have chosen the parameters so that the Lockhart model predicts a constant and common value of $\dot{\gamma}$. Fig. Bb shows that P is initially close to Lockhart predictions P^* but decreases fastly to zero; the fast decrease of P coincides with peaks of $\dot{\gamma}$ (Fig. Bc) above the value $\dot{\gamma}^*$ with a higher peak for larger values of α^a; the elastic deformation ε^e (Fig. Bd) is not constant either, with a large peak above the Lockhart-Ortega prediction for $\alpha^a = 0.9$. For all values of α^a, ε^e converges toward the threshold ε^Y.

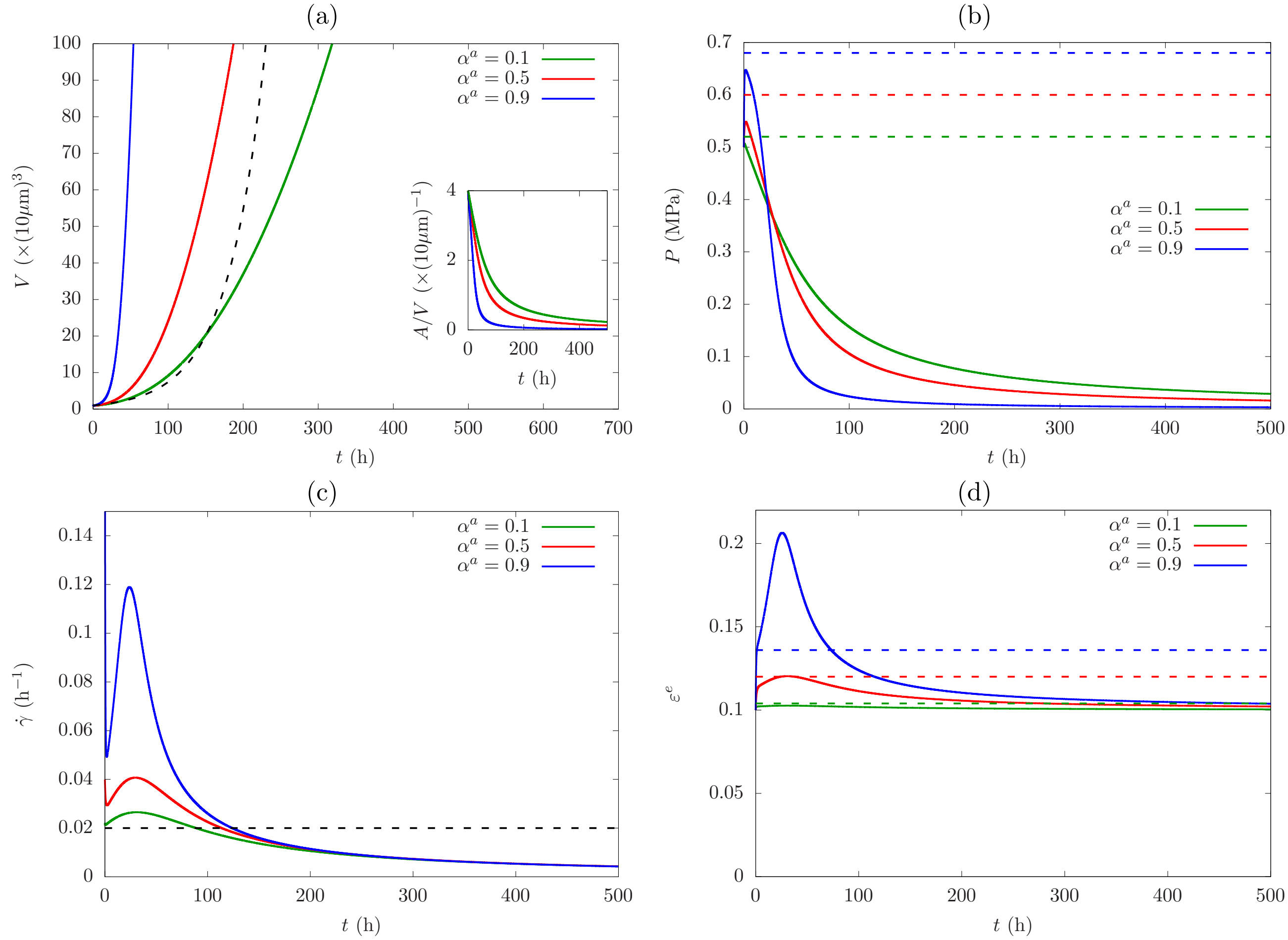

Figure B: Growth of a single hexagonal cell for three different values of α^a: time evolution of volume (inset: ratio area/volume) (a), turgor (b), relative growth rate (c), and elastic deformation of the walls (d). The dashed lines correspond to the solution of the Lockhart model; note that in the Lockhart-Ortega model, the chosen sets of parameters lead to the constant and equal value $\dot{\gamma}^* = 2\% \cdot \mathrm{h}^{-1}$, and to the same evolution of volume.
