# Supplementary material for: Coupling water fluxes with cell wall mechanics in a multicellular model of plant development
Source: PLoS Comput Biol. 2019 Jun 20;15(6):e1007121. doi: 10.1371/journal.pcbi.1007121 (PMC6605655; doi:10.1371/journal.pcbi.1007121)
Supplement: S3 Text — Detailed calculations of the explicit solution of the steady-state equations. (PDF) [file pcbi.1007121.s004.pdf]

Supplementary information for the article: Coupling water fluxes with cell wall mechanics in a multicellular model of plant development.

Calculations for the two-cells model

Ibrahim Cheddadi, Michel Génard, Nadia Bertin, Christophe Godin

Gathering the flux equation (Eq. 8 from main text), the wall mechanics equation (Eq. 1 from main text) with $\frac{\mathrm{d}P}{\mathrm{d}t} = 0$, and the fact that $\dot{\gamma}_i = \dot{\varepsilon}_i$, we get

$$\phi^a(P^M - P_0) + \frac{\phi^s}{2}(P_1 - P_0) - \phi^w(P_0 - P_0^Y)_+ = 0 \tag{S11}$$

$$\phi^a(P^M - P_1) - \frac{\phi^s}{2}(P_1 - P_0) - \phi^w(P_1 - P_1^Y)_+ = 0. \tag{S12}$$

First, we assume that both cells are growing ($P_i > P_i^Y, i = 0, 1$).

First regime: $P_i > P_i^Y, i = 0, 1$. Adding (S11) and (S12) we get:

$$\overline{P} = \alpha^a P^M + (1 - \alpha^a)\overline{P}^Y, \tag{S13}$$

where $\alpha^a = \dfrac{\phi^a}{\phi^a + \phi^w}$, $\quad \overline{P} = \dfrac{P_0 + P_1}{2}$. With Eq. 1 from main text, we get

$$\bar{\dot{\gamma}} = \frac{\phi^a \phi^w}{\phi^a + \phi^w}(P^M - \overline{P}^Y), \tag{S14}$$

where $\bar{\dot{\gamma}} = \frac{\dot{\gamma}_0 + \dot{\gamma}_1}{2}$ Therefore, the gathering of two cells behaves the same as one cell if one considers the mean values.

Then, we examine the heterogeneities in turgor and growth rate. Substracting (S11) to (S12), we get

$$\Delta P = \frac{\phi^w}{\phi^a + \phi^s + \phi^w}\Delta P^Y.$$

Let

$$\alpha^s = \frac{\phi^s}{\phi^s + \phi^a}.$$

Then the previous expression becomes

$$\Delta P = \frac{(1 - \alpha^a)(1 - \alpha^s)}{1 - \alpha^s + \alpha^a \alpha^s}\Delta P^Y. \tag{S15}$$

As $(1 - \alpha^a)(1 - \alpha^s) = 1 - \alpha^a - \alpha^s + \alpha^a\alpha^s < 1 - \alpha^s + \alpha^a\alpha^s$, we find that turgor difference ΔP cannot exceed the value ΔP^Y. When $\alpha^s = 0$ (symplasmic fluxes negligeable with respect to apoplasmic ones), then $\Delta P = (1 - \alpha^a)\Delta P^Y$; when $\alpha^s > 0$, symplasmic fluxes tend to reduce the turgor heterogeneity between cells.

With Eq. 7 from main text we get then

$$\Delta\dot{\gamma} = \frac{(\phi^a + \phi^s)\phi^w}{\phi^a + \phi^s + \phi^w}\Delta P^Y, \tag{S16}$$

where $\Delta\dot{\gamma} = \frac{\dot{\gamma}_0 - \dot{\gamma}_1}{2}$. Note that this expression is valid iff $P_1 > P_1^Y$ or equivalently $\dot{\gamma}_1 > 0$. The limit $\dot{\gamma}_1 = 0$ corresponds to the situation where cell 0 is growing in such a way that it prevents cell 1 to grow because of

the symplasmic fluxes between them. We examine how this situation can occur depending on the values of the symplasmic conductivity ϕ^s and the other parameters. We find that

$$
\begin{aligned}
P_1 > P_1^Y &\iff \frac{\phi^a + \phi^s}{\phi^a + \phi^s + \phi^w} \frac{\Delta P^Y}{P^M - \overline{P}^Y} < \frac{\phi^a}{\phi^a + \phi^w} \\
&\iff \frac{\alpha^a}{1 - (1 - \alpha^s)\alpha^a} \rho < \alpha^a \\
&\iff \alpha^s < \frac{1 - \rho}{1 - \alpha^a}.
\end{aligned}
$$

For instance, $P_0^Y = 0.25$ MPa, $P_1^Y = 0.5$ MPa, and $P^M = 0.625$ MPa yields $\rho = 0.5$. The hypothesis of this study ($P_0^Y < P_1^Y < P^M$) corresponds to the condition $\rho \in]0, 1[$. Note that if $\alpha^a > \rho$, then $\frac{1-\rho}{1-\alpha^a} > 1$, and the condition is verified whatever the value of α^s; if $\alpha^s = 1 - \rho$, the condition is equivalent to $\alpha^a > 0$, which is also always verified. Fig. 2a) in main text recapitulates the regions of the parameters space $\alpha^a \times \alpha_s$ where the condition is verified, for different values of ρ. The size of the region $\dot{\gamma}_1 = 0$ increases as ρ gets closer to 1.

Second regime: $P_0 > P_0^Y$ and $P_1 < P_1^Y$. In this case, eqs. (S11) and (S12) turn into

$$\phi^a(P^M - P_0) + \frac{\phi^s}{2}(P_1 - P_0) - \phi^w(P_0 - P_0^Y) = 0 \tag{S17}$$

$$\phi^a(P^M - P_1) - \frac{\phi^s}{2}(P_1 - P_0) = 0. \tag{S18}$$

(S18) leads to

$$P_1 = (1 - \tilde{\alpha}^s)P^M + \tilde{\alpha}^s P_0, \tag{S19}$$

where $\tilde{\alpha}^s = \frac{\phi^s}{2\phi^a + \phi^s}$. Adding eqs. (S17) and (S18) leads to

$$P_0(\phi^a + \phi^w) = 2\phi^a P^M + \phi^w P_0^Y - \phi^a((1 - \tilde{\alpha}^s)P^M + \tilde{\alpha}^s P_0),$$

then,

$$P_0(\phi^a(1 + \tilde{\alpha}^s) + \phi^w) = \phi^a(1 + \tilde{\alpha}^s)P^M + \phi^w P_0^Y,$$

and finally

$$P_0 = \alpha^{as} P^M + (1 - \alpha^{as})P_0^Y, \tag{S20}$$

where

$$\alpha^{as} = \frac{\phi^{as}}{\phi^{as} + \phi^w} \qquad \text{and} \qquad \phi^{as} = \phi^a(1 + \tilde{\alpha}^s).$$

Hence, thanks to the symplasmic fluxes from its neighbour cell 1, cell 0 benefits from an enhanced access to the apoplasmic fluxes by a factor $\phi^{as}/\phi^a = 1 + \tilde{\alpha}^s$. Then, from Eq. 1 in main text, the relative growth rate of cell 0 is

$$\dot{\gamma}_0 = \frac{\phi^{as}\phi^w}{\phi^{as} + \phi^w}(P^M - P_0^Y). \tag{S21}$$

By hypothesis, the growth rate of cell 1 is zero, and we can compute the heterogeneity in turgor: from (S19), we find that

$$\Delta P = \frac{1 - \tilde{\alpha}^s}{2}(P^M - P_0),$$

and hence

$$\Delta P = \frac{1}{2}(1 - \tilde{\alpha}^s)(1 - \alpha^{as})(P^M - P_0^Y). \tag{S22}$$
