# Supplementary material for: Coupling water fluxes with cell wall mechanics in a multicellular model of plant development
Source: PLoS Comput Biol. 2019 Jun 20;15(6):e1007121. doi: 10.1371/journal.pcbi.1007121 (PMC6605655; doi:10.1371/journal.pcbi.1007121)
Supplement: S4 Text — Detailed corresponding equations and numerical computation of the time-dependent solution. (PDF) [file pcbi.1007121.s005.pdf]

Supplementary information for the article: Coupling water fluxes with cell wall mechanics in a multicellular model of plant development.

Calculations for a chain of connected cells

Ibrahim Cheddadi, Michel Génard, Nadia Bertin, Christophe Godin

Let N be an positive integer, we consider a chain of $2N+1$ cells in series, that are connected two-by-two by their lateral faces, through which water can flow. It is a straightforward extension of the two cells model presented above, and the evolution of the cells is prescribed by the following equations:

$$\phi^a(P^M - P_i) + \frac{\phi^s}{2}(P_{i+1} + P_{i-1} - 2P_i) - \phi^w(P_i - P_i^Y)_+ = 0, \forall i = 2, \dots, 2N,$$

and

$$\phi^a(P^M - P_1) + \frac{\phi^s}{2}(P_2 - P_1) - \phi^w(P_1 - P_1^Y)_+ = 0,$$

$$\phi^a(P^M - P_{2N+1}) + \frac{\phi^s}{2}(P_{2N} - P_{2N+1}) - \phi^w(P_{2N+1} - P_{2N+1}^Y)_+ = 0.$$

As before, we have considered the case where only the yield turgor P_i^Y varies between cells.

This set of differential equations is numerically solved with the `odeint` routine from the `python` library `scipy`. The simulations are performed with a twice lower value of P_{N+1}^Y compared to the values for other cells, so that the cell at the center of the chain benefits of a mechanically favorable configuration, and we study its ability to inhibit the growth of its neighbors.

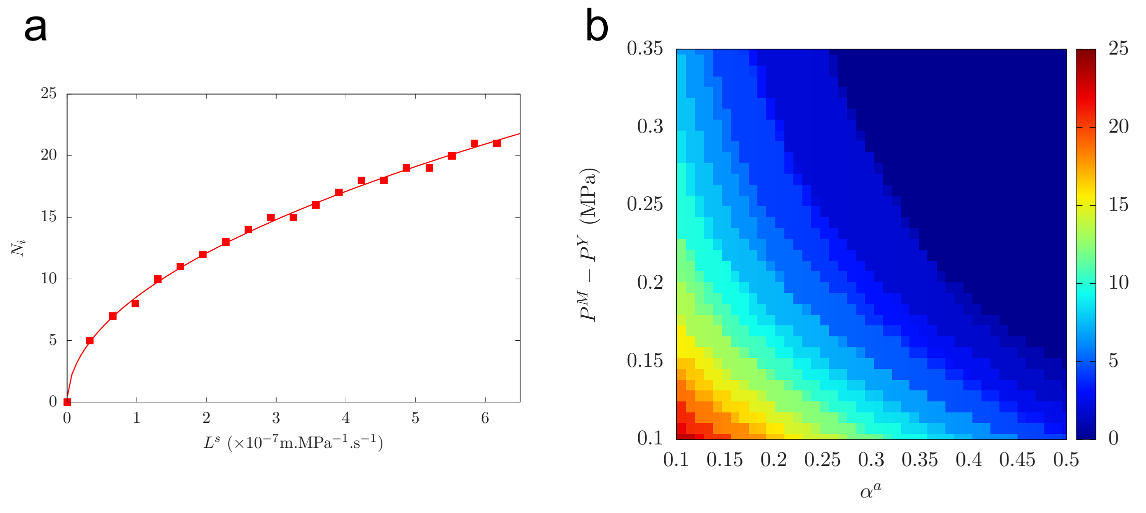

Figure A: Results for a chain of $2N+1$ cells with $N = 50$, where the central cell has twice softer walls; **a**) number N_i of cells that are inhibited on each side of the central cell, for different values of L^s; the line is a fit with a square root function, in the form $c\sqrt{L^s}$. **b**) Values of the prefactor c in the space (α^a, P^M).
