# Supplementary material for: Coupling water fluxes with cell wall mechanics in a multicellular model of plant development
Source: PLoS Comput Biol. 2019 Jun 20;15(6):e1007121. doi: 10.1371/journal.pcbi.1007121 (PMC6605655; doi:10.1371/journal.pcbi.1007121)
Supplement: S5 Text — Detailed presentation of the numerical algorithm. (PDF) [file pcbi.1007121.s006.pdf]

Supplementary information for the article: Coupling water fluxes with cell wall mechanics in a multicellular model of plant development.

Numerical resolution of the 2D multicellular model

Ibrahim Cheddadi, Michel Génard, Nadia Bertin, Christophe Godin

Structure of the mathematical problem

Thanks to the geometrical constraint of uni-directional growth, the Lockhart-Ortega is very simple to resolve. The identity between the relative growth rate of the cell and the strain rate of the walls allows to couple the equation that describes fluxes, and the equation that describes walls synthesis. Then the stress in the walls and the pressure inside the cell are linked by the mechanical equilibrium. Finally there is only one independent variable (pressure for instance) and the model can be solved analytically.

Conversely, in the bidimensionnal model we propose, the properties of a given wall (elongation rate and elastic deformation) cannot be directly linked to the properties of the adjacent cells (growth rate and pressure). Hence a new strategy has to be developped. First, we emphasize the strong coupling between fluxes and mechanics: the motion of the vertices is prescribed by the mechanical equilibrium (Eq. 12 from main text) between pressure forces and elastic forces; meanwhile, a displacement of the vertices can cause a variation of volume of several cells, which has to be balanced by water fluxes (Eq. 11 from main text); water fluxes are limited by the finite permeability of the walls, which sets a constraint on possible variations of volume. Similarly, any variation in the length of the walls leads to a modification of their elastic deformation (Eq. 7 from main text).

Another way to understand this problem is to consider it as the minimization of mechanical energy (mechanical equilibrium Eq. 12 from main text) under two constraints on the position of the vertices, through the volumes of the cells (Eq. 11 from main text) and the lengths of the edges (Eq. 7 from main text). This kind of problem is often encountered in mechanics, *e.g* solid friction, contact mechanics, or incompressible fluid mechanics; a powerfull theoritical and practical tool to solve this is the method of lagrangian multipliers. For instance, in the context of incompressible fluid mechanics, the constraint of volume conservation is relaxed by pressure that acts as a lagragian multiplier. Physically, the pressure adjusts itself so that both the constraint and the mechanical equilibrium are satisfied. The model we propose exhibits the same structure, as pressure will adjust to both fluxes and mechanical constraints. However, the system here is discrete, and the flux equation (Eq. 11 in main text) is linear with respect to pressure, so it can be reduced to a linear system. We will take advantage of this for the resolution of the model.

Resolution algorithm

Volumes and lengths as functions of the positions of the vertices. First, we express volumes and lengths as functions of the positions of the vertices. Let N_v be the number of vertices and $\boldsymbol{X} \in \mathbb{R}^{2N_v}$ the vector of the positions of all the vertices. The volume of a cell i is $V_i = S_i h$ where S_i is its surface. As cells are non intersecting polygons, their signed surface is given by the general formula

$$S_i = \frac{1}{2}\sum_{k=0}^{n_i-1}(x_k y_{k+1} - x_{k+1} y_k), \tag{S23}$$

where n_i is the number of vertices of cell i, $(x_k, y_k)_{k=0,\dots,n_i-1}$ are the coordinates of the vertices of the cell i in counterclockwise order, and we set $(x_{n_i}, y_{n_i}) = (x_0, y_0)$. Let N_c be the number of cells and $\boldsymbol{V} \in \mathbb{R}^{N_c}$ the

vector of all the cells volumes; thanks to (S23), it can be expressed as a function of $\boldsymbol{X}$ and its gradient $\nabla_{\boldsymbol{X}}\boldsymbol{V}$ with respect to $\boldsymbol{X}$ can be computed. Then the time derivative of $\boldsymbol{V}$ expresses as

$$\frac{\mathrm{d}\boldsymbol{V}}{\mathrm{d}t} = \nabla_{\boldsymbol{X}}\boldsymbol{V}\frac{\mathrm{d}\boldsymbol{X}}{\mathrm{d}t}.$$

Note here that $\nabla_{\boldsymbol{X}}\boldsymbol{V}$ is a $N_c \times 2N_e$ matrix and $\frac{\mathrm{d}\boldsymbol{X}}{\mathrm{d}t}$ is a $2N_e$ vector, so their product is well defined and has the correct dimension.

Similarly, the length of a segment k with two vertices $v_1 = (x_1, y_1)$ and $v_2 = (x_2, y_2)$ at its ends is

$$l_k = \sqrt{(x_1 - x_2)^2 + (y_1 - y_2)^2}. \tag{S24}$$

Let N_e be the number of edges and $\boldsymbol{l} \in \mathbb{R}^{N_e}$ the vector of all the edges lengths; thanks to (S24), it can be expressed as a function of $\boldsymbol{X}$ and its gradient $\nabla_{\boldsymbol{X}}\boldsymbol{l}$ with respect to $\boldsymbol{X}$ can be computed. Then the time derivative of $\boldsymbol{l}$ expresses as

$$\frac{\mathrm{d}\boldsymbol{l}}{\mathrm{d}t} = \nabla_{\boldsymbol{X}}\boldsymbol{l}\frac{\mathrm{d}\boldsymbol{X}}{\mathrm{d}t}.$$

Time discretisation. Time is discretized using a fixed time step Δt and the time derivatives are approximated by the 1st order Euler scheme, for instance:

$$\frac{\mathrm{d}\boldsymbol{X}}{\mathrm{d}t}(t) \approx \frac{\boldsymbol{X}(t+\Delta t) - \boldsymbol{X}(t)}{\Delta t}.$$

Let $\boldsymbol{\varepsilon} \in \mathbb{R}^{N_e}$ be the vector of all the elastic deformations of the edges. Let $\boldsymbol{X}^0 = \boldsymbol{X}(0)$ and $\boldsymbol{\varepsilon}^0 = \boldsymbol{\varepsilon}(0)$ be some initial conditions. We construct successive approximations of the solution at times $t_n = n\Delta t$ for $n > 0$ by solving at each time step the mechanical equilibrium (Eq. 12 from main text) along with the discretized versions of flux (Eq. 11 from main text) and wall rheology (Eq. 7 from main text) equations: let $\boldsymbol{P} \in \mathbb{R}^{N_c}$ be the vector of all the cells pressures; these equations can be written in a matrix form:

$$\nabla_{\boldsymbol{X}}\boldsymbol{V}(\boldsymbol{X}^{n+1})\frac{\boldsymbol{X}^{n+1} - \boldsymbol{X}^n}{\Delta t} = M_P\boldsymbol{P}^{n+1} + \boldsymbol{b}_P, \tag{S25}$$

$$\frac{\boldsymbol{\varepsilon}^{n+1} - \boldsymbol{\varepsilon}^n}{\Delta t} + \boldsymbol{\beta}^n\boldsymbol{\varepsilon}^{n+1} = \frac{1}{\boldsymbol{l}(\boldsymbol{X}^{n+1})}\nabla_{\boldsymbol{X}}\boldsymbol{l}(\boldsymbol{X}^{n+1})\frac{\boldsymbol{X}^{n+1} - \boldsymbol{X}^n}{\Delta t}. \tag{S26}$$

where M_P is a $N_i \times N_i$ matrix, with the following non-zero coefficients:

$$M_P(i,i) = A_i L_i^a - \sum_{j \in n(i)} A_{ij}L_{ij}^s, \qquad \forall i = 1, \ldots, N_c,$$
$$M_P(i,j) = A_{ij}L_{ij}^s, \quad \forall i = 1, \ldots, N_c, \qquad \forall j \in n(i),$$

with $\boldsymbol{b}_P \in \mathbb{R}^{N_c}$ is defined by its coefficients

$$\boldsymbol{b}_p(i) = A_i L_i^a P^M, \qquad \forall i = 1, \ldots, N_c.$$

Note here that the model implies no time derivative of the pressure, so that $\forall n > 0$, $\boldsymbol{P}^{n+1}$ can be computed without the knowledge of $\boldsymbol{P}^n$, and the initial value of the pressure is not needed.

In addition, $\boldsymbol{\beta}^n$ is the $N_e \times N_e$ diagonal matrix with components $\boldsymbol{\beta}^n(k,k) = \frac{2w}{h}\phi_k^w E_k \max\left(0, \frac{\varepsilon_k^n - \varepsilon_k^Y}{\varepsilon_k^n}\right)$ for $k = 1, \ldots, N_e$, and for the purpose of notation, $\frac{1}{\boldsymbol{l}}$ is the $N_e \times N_e$ diagonal matrix with components $1/l_k$. Note here that the variables $\boldsymbol{\beta}^n$ are taken at time step n so that they are considered as constants at time step $n+1$ and the equation (S26) is linear with respect to the unknown $\boldsymbol{\varepsilon}^{n+1}$.

Pressure and elastic deformation as functions of the position of the vertices. Thanks to this time discretization, we see that at each time step, the unknown pressure $\boldsymbol{P}^{n+1}$ and elastic deformation $\boldsymbol{\varepsilon}^{n+1}$ are defined through the linear equations (S25) and (S26) which can be easily inverted, which allows to express both these variables as functions of the spatial unknown $\boldsymbol{X}^{n+1}$.

First, from equation (S25):

$$P(\boldsymbol{X}^{n+1}) = \frac{1}{\Delta t}M_P^{-1}\nabla_{\boldsymbol{X}}\boldsymbol{V}(\boldsymbol{X}^{n+1})\boldsymbol{X}^{n+1} - M_P^{-1}\left(\frac{1}{\Delta t}\nabla_{\boldsymbol{X}}\boldsymbol{V}(\boldsymbol{X}^{n+1})\boldsymbol{X}^n - \boldsymbol{b}_P\right). \tag{S27}$$

Then, using (S26):

$$\boldsymbol{\varepsilon}\left(\boldsymbol{X}^{n+1}\right)=\frac{1}{\Delta t}M_{\varepsilon}^{-1}\frac{1}{\boldsymbol{l}(\boldsymbol{X}^{n+1})}\nabla_{\boldsymbol{X}}\boldsymbol{l}(\boldsymbol{X}^{n+1})\boldsymbol{X}^{n+1}-\frac{1}{\Delta t}M_{\varepsilon}^{-1}\left(\frac{1}{\boldsymbol{l}(\boldsymbol{X}^{n+1})}\nabla_{\boldsymbol{X}}\boldsymbol{l}(\boldsymbol{X}^{n+1})\boldsymbol{X}^{n}-\boldsymbol{\varepsilon}^{n}\right), \tag{S28}$$

where $M_{\varepsilon}=\frac{1}{\Delta t}I_{N_e}+\boldsymbol{\beta}^n$.

Structure of the resolution algorithm Thanks to the two previous steps, we are now able to propose a algorithm for the resolution of the model.

- Initialization: Define $\boldsymbol{X}^0\in\mathbb{R}^{2N_v}$ and $\boldsymbol{\varepsilon}^0\in\mathbb{R}^{N_e}$
- $\forall n\geq 0$, assuming $\boldsymbol{X}^n$ and $\boldsymbol{\varepsilon}^n$ are known, let $\boldsymbol{F}^n:\mathbb{R}^{2N_v}\to\mathbb{R}^{2N_v}$ be the function such that $\forall v=0,\ldots,N_v-1$,

$$\begin{pmatrix} F^n_{2v+1}(\boldsymbol{X})\\ F^n_{2v+2}(\boldsymbol{X})\end{pmatrix}=\frac{1}{2}\sum_{k\in f(v)}\Delta_k P(\boldsymbol{X})\ A_k(\boldsymbol{X})\boldsymbol{n}_k(\boldsymbol{X})+\sum_{k\in f(v)}E_k\varepsilon^e_k(\boldsymbol{X})a_k(\boldsymbol{X})\boldsymbol{e}_{k,v}(\boldsymbol{X}),$$

  where F^n_k is the k-th component of $\boldsymbol{F}^n$, and with the same notations as in Eq. 12 from main text; $\boldsymbol{P}(\boldsymbol{X})$ and $\boldsymbol{\varepsilon}(\boldsymbol{X})$ are the functions of $\boldsymbol{X}$ given by (S27) and (S28). Then, the new position of the vertices $\boldsymbol{X}^{n+1}$ is the solution of the equation

$$\boldsymbol{F}^n(\boldsymbol{X})=0. \tag{S29}$$

Resolution of (S29)**.** This is the last and most critical step of the resolution algorithm. The problem of computing the roots of a multidimensional non linear function is often encountered in the mechanical modelling of complex multibody systems, and a method of choice for the resolution is the Newton algorithm [1]. It is a iterative process which derives from a Taylor expansion about a current point $\boldsymbol{u}^k$:

$$\boldsymbol{F}^n(\boldsymbol{u}^{k+1})=\boldsymbol{F}^n(\boldsymbol{u}^k)+J(\boldsymbol{u}^k)(\boldsymbol{u}^{k+1}-\boldsymbol{u}^k)+o(\boldsymbol{u}^{k+1}-\boldsymbol{u}^k),$$

where $J(\boldsymbol{u}^k)$ is the jacobian matrix of function $\boldsymbol{F}^n$. The new value $\boldsymbol{u}^{k+1}$ is obtained by setting the right-hand side to zero and neglecting the high order term, and then solving the linear system:

$$J(\boldsymbol{u}^k)\delta\boldsymbol{u}^k=-F^n(\boldsymbol{u}^k),\boldsymbol{u}^{k+1}=\boldsymbol{u}^k+\delta\boldsymbol{u}^k.$$

With the initial value $\boldsymbol{u}^0=\boldsymbol{X}^n$, iterations are run until a stopping criterium is met, for instance

$$\frac{\|\boldsymbol{F}^n(\boldsymbol{u}^k)\|}{\|\boldsymbol{F}^n(\boldsymbol{u}^0)\|}\leq tol_{res}, \tag{S30}$$

where $tol_{res}>0$ is a fixed value. Then one can set $\boldsymbol{X}^{n+1}=\boldsymbol{u}^k$.

The compution of the jacobian matrix $J(\boldsymbol{u}^k)$ is non trivial here because of the numerous non-linearities of function $\boldsymbol{F}^n$. Therefore we have chosen to use the Newton-Krylov variant of this algorithm, that avoids the computation of the jacobian without loosing efficiency [1].

However, Newton methods in general have only local convergence properties, which means that they need an initial guess close enough to the solution to be able to converge. This is critical for instance in the first time step of the simulation, because the initial conditions might be far from equilibrium, but also for further time steps. This lack of global convergence properties is often dealt with by adding a friction term proportional to the velocity and hence to the time derivative of the positions. With this method, the problem to solve at each time step becomes after time discretization: find $\boldsymbol{X}$ such that

$$\boldsymbol{G}(\boldsymbol{X})=\boldsymbol{F}^n(\boldsymbol{X})-c\frac{\boldsymbol{X}-\boldsymbol{X}^n}{\Delta t}=0,$$

where $c>0$ is a friction coefficient. This new problem is easier to solve with the Newton method, all the more that c is large. However, the root of $\boldsymbol{G}$ might not satisfy the condition (S30), and in addition its value depends on the value of c. Therefore, instead of applying the Newton method to the function $\boldsymbol{G}$, we perform the following iterative process:

- Initialization: $\boldsymbol{u}^0=\boldsymbol{X}^n$

- Assuming $\boldsymbol{u}^k$ is known, compute $\boldsymbol{u}^{k+1}$ as the solution of

$$\boldsymbol{G}^k(\boldsymbol{u}^{k+1}) = 0, \tag{S31}$$

where $\boldsymbol{G}^k(\boldsymbol{u}^{k+1}) = \boldsymbol{F}^n(\boldsymbol{u}^{k+1}) - c^k \frac{\boldsymbol{u}^{k+1}-\boldsymbol{u}^k}{\Delta t}$, and the value $c^k > 0$ will be adjusted to ensure a robust convergence (see below). This solution is computed thanks to the Newton method, with the tolerance $tol_{res}/10$ in the stopping criterium.

- The iterations are stopped when $\frac{\|\boldsymbol{F}^n(\boldsymbol{u}^k)\|}{\|\boldsymbol{F}^n(\boldsymbol{u}^0)\|} \leq tol_{res}$. Then the choice $\boldsymbol{X}^{n+1} = \boldsymbol{u}^k$ is an approximate solution of (S29).

In this algorithm, the choice of the friction coefficient c^k is not straightforward: a large value would ensure the convergence of subproblem (S31), but it would also slow down the convergence toward the solution of problem (S29). To avoid this, we choose a large initial value c^0 and decrease it with the law $c^{k+1} = c^k/2$. This choice ensures a robust behaviour of the algorithm.

References

1. Knoll DA, Keyes DE. Jacobian-free Newton-Krylov methods: a survey of approaches and applications. J Comp Phys. 2004;193:357–397.
