# Supplementary material for: Coupling water fluxes with cell wall mechanics in a multicellular model of plant development
Source: PLoS Comput Biol. 2019 Jun 20;15(6):e1007121. doi: 10.1371/journal.pcbi.1007121 (PMC6605655; doi:10.1371/journal.pcbi.1007121)
Supplement: S6 Text — Detailed explanations for the choice and definitions of the parameters sets used for the bump simulations. (PDF) [file pcbi.1007121.s007.pdf]

Supplementary information for the article: Coupling water fluxes with cell wall mechanics in a multicellular model of plant development.

Parameters used for the bump simulations

Ibrahim Cheddadi, Michel Génard, Nadia Bertin, Christophe Godin

Let R_0 be the initial radius of the cell, then $P^Y = \frac{w}{R_0 \cos(\pi/6)} E\varepsilon^Y$ is a representative value for the yield turgor of a hexagonal cell. However we have observed that the effective threshold pressure is approximately twice lower in multicellular tissues and we have adapted the value of E accordingly: we choose E such that $P^Y = 0.5$ MPa and multiplied this value by two to obtain a an order of magnitude for the initial turgor of the cell close to the target value 0.5 MPa. The value $\varepsilon^Y = 0.1$ is chosen accordingly to experimental observations where wall deformations can be of the order of 10%. We choose two values for P^M: 0.55 MPa close to the threshold, and 0.7 MPa. Finally, we can use the Lockhart's prediction $\dot{\gamma}^*$ (Eq.6 from main text) as an order of magnitude of the relative growth rate; we choose $\dot{\gamma}^* = 2\% \cdot \mathrm{h}^{-1}$. Then, a given value of α^a (evaluated with $R = R_0$) sets a unique value of ϕ^a and ϕ^w. Then we obtain $\Phi^w = \frac{2w}{h}\phi^w$, and the value of ϕ^a and α^s yields the value of ϕ^s. From ϕ^a and ϕ^s we finally obtain the values of L^a and L^s. The table A recapitulates the sets of parameters used in this article, either with the control parameters

$$\varepsilon^Y, P^M, P^Y, \dot{\gamma}^*, \alpha^a, \alpha^s \tag{S32}$$

or equivalently with the actual parameters of the model

$$\varepsilon^Y, P^M, E, \Phi^w, L^a, L^s. \tag{S33}$$

The correspondance has been obtained with $R_0 = 6.5$ μm, $h = 10$ μm and $w = 0.5$ μm.

Table A: Parameters used for the bump simulation (see Fig. 3 in main text). The top part of the table refers to the control parameters (S32), and the bottom part to the actual parameters (S32) used in the 2D model. The rightmost parameters after the vertical double bar are specific to multicellular models as they quantify the water conductivity between neighbour cells. The geometrical parameters are $h = 10\mu$m and $w = h/20$.

**Control parameters**	ε^Y	P^M (MPa)	P^Y_6 (MPa)	$\dot{\gamma}^*$ (h^{-1})	α^a	α^s
(REF)	0.1	0.7	0.5	$2 \cdot 10^{-2}$	0.1	0.9
(CC-)	0.1	0.7	0.5	$2 \cdot 10^{-2}$	0.1	0.1
(ALPHA+)	0.1	0.7	0.5	$2 \cdot 10^{-2}$	0.9	0.9
(PM-)	0.1	0.55	0.5	$0.5 \cdot 10^{-2}$	0.1	0.9
**Actual parameters**	ε^Y	P^M (MPa)	E (MPa)	Φ^w ($\mathrm{MPa}^{-1}.\mathrm{s}^{-1}$)	L^a ($\mathrm{m.MPa}^{-1}.\mathrm{s}^{-1}$)	L^s ($\mathrm{m.MPa}^{-1}.\mathrm{s}^{-1}$)
(REF)	0.1	0.7	112.6	$2.8 \cdot 10^{-5}$	$8.7 \cdot 10^{-11}$	$7.8 \cdot 10^{-10}$
(CC-)	0.1	0.7	112.6	$2.8 \cdot 10^{-5}$	$8.7 \cdot 10^{-11}$	$9.6 \cdot 10^{-12}$
(ALPHA+)	0.1	0.7	112.6	$3.1 \cdot 10^{-6}$	$7.8 \cdot 10^{-10}$	$7.0 \cdot 10^{-9}$
(PM-)	0.1	0.55	112.6	$2.8 \cdot 10^{-5}$	$8.7 \cdot 10^{-11}$	$7.8 \cdot 10^{-10}$
